# Immunoglobulin gene analysis in chronic lymphocytic leukemia in the era of next generation sequencing

**DOI:** 10.1038/s41375-020-0923-9

**Published:** 2020-06-19

**Authors:** Frédéric Davi, Anton W. Langerak, Anne Langlois de Septenville, P. Martijn Kolijn, Paul J. Hengeveld, Anastasia Chatzidimitriou, Silvia Bonfiglio, Lesley-Ann Sutton, Richard Rosenquist, Paolo Ghia, Kostas Stamatopoulos, on behalf of ERIC, the European Research Initiative on CLL, and the EuroClonality-NGS Working Group

**Affiliations:** 1 Department of Hematology, APHP, Hôpital Pitié-Salpêtrière and Sorbonne University, Paris, France; 2 Department of Immunology, Laboratory Medical Immunology, Erasmus MC, Rotterdam, The Netherlands; 3 Institute of Applied Biosciences, Centre for Research and Technology Hellas, Thessaloniki, Greece; 4 Division of Experimental Oncology, Università Vita-Salute San Raffaele and IRCCS Ospedale San Raffaele, Milan, Italy; 5 Department of Molecular Medicine and Surgery, Karolinska Institutet, Stockholm, Sweden; 6 Clinical Genetics, Karolinska University Laboratory, Karolinska University Hospital, Stockholm, Sweden

**Keywords:** Cancer genetics, B-cell receptor

## Abstract

Twenty years after landmark publications, there is a consensus that the somatic hypermutation (SHM) status of the clonotypic immunoglobulin heavy variable (IGHV) gene is an important cornerstone for accurate risk stratification and therapeutic decision-making in patients with chronic lymphocytic leukemia (CLL). The IGHV SHM status has traditionally been determined by conventional Sanger sequencing. However, NGS has heralded a new era in medical diagnostics and immunogenetic analysis is following this trend. There is indeed a growing demand for shifting practice and using NGS for IGHV gene SHM assessment, although it is debatable whether it is always justifiable, at least taking into account financial considerations for laboratories with limited resources. Nevertheless, as this analysis impacts on treatment decisions, standardization of both technical aspects, and data interpretation becomes essential. Also, the need for establishing new recommendations and providing dedicated education and training on NGS-based immunogenetics is greater than ever before. Here we address potential and challenges of NGS-based immunogenetics in CLL. We are convinced that this perspective helps the hematological community to better understand the pros and cons of this new technological development for CLL patient management.

## Background

Twenty years after landmark publications by the Chiorazzi and Stevenson groups [1, 2], there is a consensus that the somatic hypermutation (SHM) status of the clonotypic immunoglobulin heavy variable (IGHV) gene is one of the cornerstones for accurate risk stratification of patients with chronic lymphocytic leukemia (CLL), which is pivotal for the realization of precision medicine in this still incurable disease. Indeed, patients with a significant SHM imprint (IGHV-mutated, M-CLL) experience a considerably more indolent disease course compared to those with limited or no SHM (IGHV-unmutated, U-CLL), who generally progress faster and have a shorter overall survival [1, 2].

IGHV gene SHM status is one of the most robust prognostic markers in CLL, readily identifiable at diagnosis and independent of clinical stage or other biomarkers. More importantly, it remains stable over time, thus contrasting all other well-established prognostic markers, including genomic aberrations, which are affected by or reflect disease evolution [3]. Furthermore, IGHV gene SHM status has a strong predictive value for response to treatment, i.e., U-CLL displays shorter progression-free survival after chemoimmunotherapy with the fludarabine, cyclophosphamide, and rituximab regimen compared to M-CLL, whereas U-CLL respond more favorably to ibrutinib-based treatment than to chemoimmunotherapy [4]. The importance of determining IGHV gene SHM status for clinical decision-making was highlighted by the firm recommendation in the most recent International Workshop on CLL guidelines to perform this test prior to treatment initiation in all patients with CLL, i.e., both in general practice and in clinical trials [5].

The European Research Initiative on CLL (ERIC) (www.ericll.org) has been at the forefront of establishing initiatives related to IG gene sequence analysis, particularly with regards to promoting good practices, while also ensuring the widest possible dissemination globally. ERIC accomplishments in this field include: (1) establishment of the ERIC IG Network (http://www.ericll.org/ignetwork/), which aims to promote awareness throughout the hematological community about the need to apply standardized and consistent analytical methods, based on the state-of-the-art in immunogenetics and the most innovative bioinformatics tools: the ERIC IG Network currently comprises 7 European reference labs and more than 100 labs across five continents, certified by ERIC for performing accurate immunogenetic analysis in CLL; (2) organization of educational events that combine lectures, computer-based practical sessions, interactive discussions, and expert panel-led debates on topics pertinent to immunogenetics in CLL; (3) providing the community with an online expert forum (http://www.ericll.org/pages/submission_form) to discuss general queries on IG gene sequence interpretation in CLL or to analyze and provide advice about complex IG gene rearrangement sequences that can be difficult to interpret in everyday practice; (4) creation and maintenance of the ERIC-IMGT/CLL-DB (http://www.imgt.org/CLLDBInterface/query), the largest database of IGHV–IGHD–IGHJ gene rearrangement sequences from patients with CLL, currently holding sequence data from over 32,000 patients collected from 33 institutions spanning Europe, the USA, and further afield; (5) frequent publication of recommendations for the determination of IGHV gene SHM status in CLL, complemented by instructions detailing how to handle analytically challenging cases or cases difficult to categorize [6, 7]. These recommendations have been widely adopted and cited by the scientific community and assist in standardizing methodologies and ultimately ensuring the acquisition of robust results.

## Next generation sequencing for immunogenetic analysis in CLL: rationale for its use and potential added value

Historically, immunogenetic analysis in CLL has been performed using low-throughput (Sanger-based) methodologies. These approaches provide an accurate and unambiguous result in the vast majority of CLL cases, likely due to the fact that CLL is dominated by a single clonal expansion (Table 1). Hence, a pertinent question is, why the need for alternative, high-throughput approaches?

**Table 1:** Comparison of Sanger sequencing vs. NGS for IGHV gene SHM analysis.

	Sanger sequencing	NGS
Pros	Relatively cheap	More detailed insight into subclonal architecture and intraclonal diversity
	Relatively short TAT (1–2 weeks)*	Combination with other assays in one NGS workflow
	Adapted to small number of cases	Adapted to large number of cases
	Data interpretation mostly straightforward and highly standardized	Circumvents need of laborious (cloning) techniques in cases with biallelic rearrangements
Cons	No insight into subclonal architecture and intraclonal diversity	(Still) relatively costly (although depending on the number of samples analyzed simultaneously)
	No combination with other assays in one workflow possible	Longer TAT (>2 weeks)*
		Need of dedicated bioinformatics tools
		Data interpretation more complicated and not (yet) standardized

*TAT* turn around time.

*TAT is highly dependent on the number of analyzed samples (batch efficiency).

First, although Sanger-based immunogenetic analysis is straightforward in the vast majority of CLL patients, it is not universally successful. Indeed, in 3–4% of cases, this analysis may either fail completely or produce results that are impossible to interpret [e.g., single unproductive rearrangement or double productive rearrangements with discordant SHM status, to name but a few [6]], thus hindering clinical decision-making, since IGHV SHM status is predictive of response to different treatment modalities and guides therapeutic decision-making. Fundamental to most causes of concern is the enormous potential diversity of IG gene rearrangements, necessitating the use of multiplex PCR approaches with consensus primers that are always a compromise.

Second, low-throughput approaches are inherently limited with regards to accurately characterizing: (1) the clonal composition (co-existence of minor clones along with the dominant clone) of a given case and the intraclonal temporal dynamics (clonal drift, previously reported to occur in CLL) [8]; and, (2) the subclonal “architecture” essentially arising from intraclonal diversification of the IG genes in the context of ongoing SHM that may lead to extensive “branching” of the clone [9, 10] with as yet unknown prognostic implications.

Third, the times are a-changing. Technological developments have paved the way for a paradigm shift in clinical diagnostics with an ever-increasing number of diagnostic laboratories adopting next generation sequencing (NGS) into their existing workflows. With the introduction of NGS for immunogenetics analysis (collectively termed Repertoire Sequencing (RepSeq)), deeper investigations of IG (and similarly T-cell receptor (TR)) gene rearrangements are now within reach, which could have a profound impact on all applications of immunogenetic analysis, including IGHV gene SHM analysis in CLL [11].

In essence, NGS could offer solutions to the analytical problems mentioned above and, moreover, assist in addressing open issues in immunogenetic analysis in CLL (Table 1). Therefore, NGS holds the potential to offer new knowledge of both biological and clinical relevance for improved understanding and management of CLL.

Besides the abovementioned considerations there is another frequently used argument for switching from classical Sanger-based analysis to NGS. Now that an increasing number of molecular diagnostic assays are transformed into NGS-based protocols, it could be very advantageous to combine multiple assays into a general NGS workflow. In the case of CLL, the need to have reliable assays for IGHV gene SHM analysis and IGH marker identification for minimal residual disease (MRD) purposes could in principle be combined via an IGH leader-based NGS strategy. This combination of assays could be extended such that the IGHV gene SHM status as well as the mutational status of several (onco)genes, such as *TP53*, but potentially also including *NOTCH1*, *ATM*, *SF3B1* [when and if these biomarkers will be shown to carry a value for decision-making in CLL], could be obtained simultaneously in a single NGS sequencing run. Indeed, libraries specific for IGHV-IGHD-IGHJ gene rearrangements and a panel of genes meaningful for CLL prognostication or treatment decision can be prepared independently and then pooled for combined sequencing on the same run. An appropriate ratio of these libraries will however need to be determined in order to ensure a sequencing depth appropriate for each gene. On the one hand, the sequencing depth and coverage should be sufficient to facilitate the detection of minor oncogenic mutations, such as those occurring within the *TP53* gene, which may influence disease evolution and treatment decisions, while on the other hand attaining deep sequencing is less relevant for the determination of IGHV gene SHM status due to the size of the dominant CLL clone. Generally speaking, such combined workflows would be very attractive, both from a laboratory organization as well as a cost-efficiency perspective.

## Potential pitfalls and issues when considering NGS-based approach for IGHV SHM status analysis in CLL

No matter how attractive and promising NGS-based immunogenetic analysis in CLL could be, there are several technical pitfalls and biological questions, which deserve attention before NGS-based immunogenetics can be safely implemented within routine diagnostic laboratory testing.

### Technical pitfalls

#### Amplification bias and quantitation issues

An interesting feature of NGS is that it provides quantitative results, e.g., one can determine clonotype size based on read numbers [11]. However, one should be careful when extrapolating this to cellular clone size, as most of the currently used methods are PCR based. These come with amplification biases that can result in distortion of clone representation, an issue that should be carefully evaluated and taken into consideration. This is particularly true when biallelic IGHV–IGHD–IGHJ gene rearrangements are present in a CLL clone (typically one being productive, the other unproductive) [6], where unbalanced clonotype size may be difficult to interpret.

#### Lack of standardized and multicenter validated protocols

A major concern for using NGS-based methods for clinical applications, such as the determination of IGHV gene SHM status in CLL, pertains to the robustness of the methodology. This is essential, considering that this biomarker is not only prognostically relevant but also predictive [4, 12, 13], and is being utilized more and more to guide therapeutic decisions. Therefore, both the wet lab workflow, relating to library preparation, and the bioinformatics data analysis have to be highly accurate and reproducible. This clearly requires standardized protocols, which should be validated in a multicenter fashion. Although commercial kits exist, to our knowledge their performance in a multicenter approach has not yet been demonstrated. The ERIC IG network and EuroClonality-NGS Working Group are currently collectively working on this.

#### Need for dedicated bioinformatics tools

Robust IG/TR gene analysis with NGS critically relies on the availability of dedicated bioinformatics tools. In contrast to other genes, one cannot use tools based on simple comparison with a reference genome. This is due to the fact that: (1) antigen receptor variable domains are created by the assembly of 2 (V and J) or 3 (V, D, and J) types of genes, and, (2) random nucleotides are deleted and/or inserted at the junctions between these genes, thus resulting in a huge variability of sequences. With the growing interest for immune RepSeq and multiple applications in various scientific and/or medical fields, there has been an intense development of bioinformatics tools designed to address all different issues related to this topic. However, most of these developments stem from research laboratories and require extensive expertise in bioinformatics, which is often limited in clinical laboratories. Moreover, to fully enter the clinical arena, bioinformatics tools need to be compliant with all the requirements of quality assessment schemes, including, to name a few, security issues related to patient data transfer and storage, software maintenance and upgrades.

### Biological open issues

#### Deeper resolution: CLL malignant clone vs. immunereactive clone

Most Sanger-based protocols rely on direct sequencing, where only the most prevalent sequence appears on the pherograms, provided that the cell population is monoclonal and bears only a monoallelic rearrangement as demonstrated by GeneScan analysis. NGS-based assays offer a far better resolution and may depict a much more complex reality.

**Fig. 1:**
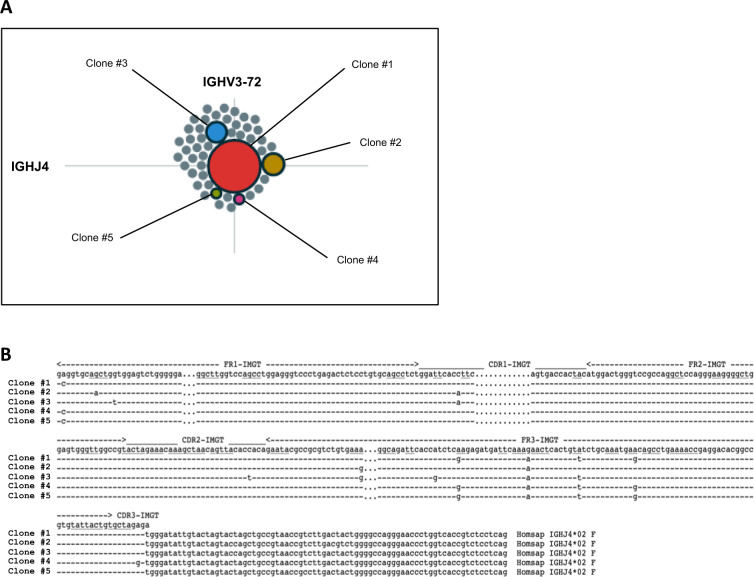
Intraclonal diversity apparent with NGS analysis. **a** Vidjil display of sequences obtained from the clonotypic IGHV/IGHD/IGHJ gene rearrangement of a CLL case. All sequences correspond to the same IGHV3-72/IGHD2-2/IGHJ4 gene rearrangement and are grouped according to their identity. The size of each “bubble” reflects the sequence abundance, resulting in a dominant clonotype surrounded by multiple “satellite” minor variant clonotypes. **b** Nucleotide sequence alignment by IMGT/V-QUEST of the five most abundant clonotypes showing evidence of intraclonal diversity. Nucleotide variants within the VH CDR3 are boxed. Clonotype frequencies: clone 1, 35.2%; clone 2, 2.4%; clone 3, 1.5%; clone 4, 0.15%; clone 5, 0.13%.

First, sequence variations of the tumor clonotype will become obvious, even if these differences account for only a minor proportion of sequences, whereas they were, mostly not detected by the low-throughput direct Sanger sequencing technology (Fig. 1). How much of this variation is artifactual due to PCR and sequencing errors or, indeed, reflective of true biological intraclonal diversity resulting from ongoing in vivo SHM remains to be determined [14–16]. Of note, such intraclonal diversity has been previously reported [9, 10], but was based on laborious cloning methods, thus certainly underestimating the extent of this phenomenon, which clinical significance, if any, warrants further studies.

**Fig. 2:**
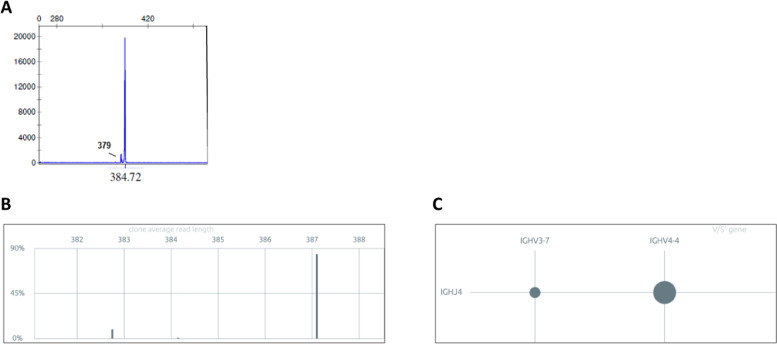
Oligoclonality. **a** GeneScan profiling of a CLL case showing a dominant clonal peak (385 bp) but also a very minor one (379 bp). Using Sanger sequencing, only the dominant clonal IGHV/IGHD/IGHJ gene rearrangement would be characterized. By contrast, NGS offers the possibility of providing sequence data for both rearrangements, as shown in these two types of visualization by Vidjil: either by “GeneScan-like” clonotype size (**b**), or by IGHV and IGHJ gene composition (**c**). The dominant clonotype (84.5% of all sequences) corresponds to a mutated (88.5% germline identity) IGHV4-4/IGHD1-26/IGHJ4 gene rearrangement, while the minor one (9.3% of all sequences) corresponds to a mutated (93.1% germline identity) IGHV3-7/IGHD3-16/IGHJ4 gene rearrangement. Both rearrangements are productive.

Second, the higher resolution afforded by NGS methodology will offer the possibility to detect independent, unrelated clonotypes that emerge above what can be considered as “polyclonal background.” Such clonotypes may go undetected by Sanger direct sequencing due to either their small size or inefficient amplification (Fig. 2). Relevant to mention in this respect, a recent study reported such multiple clones in almost one quarter of CLL cases, and furthermore proposed that their presence had prognostic value [17]. While such findings have to be confirmed, they underline the need to better define rules and limits for what constitutes a “molecular clone.”

## Toward implementation of NGS-based determination of IGHV gene SHM status in routine diagnostics

NGS immunogenetics is the focus of the EuroClonality-NGS Working Group (https://euroclonalityngs.org/usr/pub/pub.php), which was launched in 2012 and consists of several EuroClonality laboratories experienced in designing assays for detecting IG/TR rearrangements, supplemented by laboratories with expertise in IG/TR gene-based MRD studies, IG/TR (clonal) repertoire studies, immunoinformatics, and bioinformatics. In recent years, ERIC and the EuroClonality-NGS Working group have been collaborating systematically on the development of a robust pipeline for NGS-based determination of SHM status in CLL for application with in a routine diagnostic setting, covering both in vitro and in silico aspects. The former pertains to amplification and sequencing while the latter concerns novel bioinformatics solutions that would be “user-friendly” (based on a web interface with no requirement for in-depth computational expertise by the user), offer intuitive graphic visualization of the results and also operate with reasonable speed compatible so as to meet the time-sensitive requirements of clinical reporting. This joint initiative has reached a mature stage, whereby the developed end-to-end pipeline is tested and refined by expert laboratories from both organizations.

ERIC and the EuroClonality-NGS Working Group are also placing great emphasis on the correct interpretation of the obtained results. Capitalizing on the availability of well-annotated primary patient samples, facilitated by the ERIC database, a standardized registration system holding clinicobiological information from patients with CLL (http://www.ericll.org/ongoing-projects/), we are seeking to generate sufficient data that will shed light on open issues regarding both the biological and clinical implications of the findings, more specifically: the true meaning of minor expanded clonotypes unrelated to the dominant one; the extent and clinical impact of intraclonal diversification; reviewing the cutoffs for discriminating U-CLL from M-CLL and determining whether there is a need for setting novel clinically relevant germline identity cutoffs.

## Conclusions

NGS has heralded a new era in medical diagnostics and immunogenetic analysis is following this trend. There is indeed a growing demand for shifting practice and using NGS for IGHV gene SHM assessment, although it is debatable whether it is always justifiable, at least taking into account financial considerations for laboratories with limited resources. While NGS will probably become the method of choice, the traditional Sanger sequencing is still the standard method for IGHV gene SHM assessment. Nevertheless, as this analysis impacts on treatment decisions, standardization of both technical aspects and data interpretation becomes even more essential. Therefore, the need for establishing new recommendations and providing dedicated education and training on NGS-based immunogenetics is greater than ever before.
